# Oral adenosine-5’-triphosphate (ATP) administration increases blood flow following exercise in animals and humans

**DOI:** 10.1186/1550-2783-11-28

**Published:** 2014-06-13

**Authors:** Ralf Jäger, Michael D Roberts, Ryan P Lowery, Jordan M Joy, Clayton L Cruthirds, Christopher M Lockwood, John A Rathmacher, Martin Purpura, Jacob M Wilson

**Affiliations:** 1 Increnovo LLC, 2138 E Lafayette Pl, Milwaukee, WI 53202, USA; 2 School of Kinesiology, Molecular and Applied Sciences Laboratory, Auburn University, Auburn, AL 36849, USA; 3 Department of Health Sciences and Human Performance, The University of Tampa, 318 N Boulevard, Tampa, FL 33606, USA; 4 Department of Biomedical Sciences, College of Veterinary Medicine, University of Missouri-Columbia, 1600 Rollins, Columbia, MO 65211, USA; 5 4Life Research, 9850 S 300 W, Sandy, UT 84070, USA; 6 Metabolic Technologies Inc., 2711 S Loop Dr, Suite 4400, Ames, IA 50010, USA; 7 Department of Animal Sciences, Iowa State University, Ames, IA, 50011, USA

## Abstract

**Introduction:**

Extracellular adenosine triphosphate (ATP) stimulates vasodilation by binding to endothelial ATP-selective P2Y2 receptors; a phenomenon, which is posited to be accelerated during exercise. Herein, we used a rat model to examine how different dosages of acute oral ATP administration affected the femoral blood flow response prior to, during, and after an exercise bout. In addition, we performed a single dose chronic administration pilot study in resistance trained athletes.

**Methods:**

Animal study: Male Wistar rats were gavage-fed the body surface area, species adjusted human equivalent dose (HED) of either 100 mg (n=4), 400 mg (n=4), 1,000 mg (n=5) or 1,600 mg (n=5) of oral ATP as a disodium salt (Peak ATP®, TSI, Missoula, MT). Rats that were not gavage-fed were used as controls (CTL, n=5). Blood flow was monitored continuously: a) 60 min prior to, b) during and c) 90 min following an electrically-evoked leg-kicking exercise. Human Study: In a pilot study, 12 college-aged resistance-trained subjects were given 400 mg of ATP (Peak ATP®, TSI, Missoula, MT) daily for 12 weeks, and prior to an acute arm exercise bout at weeks 1, 4, 8, and 12. Ultrasonography-determined volumetric blood flow and vessel dilation in the brachial artery was measured at rest, at rest 30 minutes after supplementation, and then at 0, 3, and 6 minutes after the exercise.

**Results:**

Animal Study: Rats fed 1,000 mg HED demonstrated significantly greater recovery blood flow (p < 0.01) and total blood flow AUC values (p < 0.05) compared to CTL rats. Specifically, blood flow was elevated in rats fed 1,000 mg HED versus CTL rats at 20 to 90 min post exercise when examining 10-min blood flow intervals (p < 0.05). When examining within-group differences relative to baseline values, rats fed the 1,000 mg and 1,600 mg HED exhibited the most robust increases in blood flow during exercise and into the recovery period. Human study: At weeks 1, 8, and 12, ATP supplementation significantly increased blood flow, along with significant elevations in brachial dilation.

**Conclusions:**

Oral ATP administration can increase post-exercise blood flow, and may be particularly effective during exercise recovery.

## Background

Adenosine-5′-triphosphate (ATP) is involved in all aspects of biosynthesis in cells and acts as the primary intracellular energy source. Extracellular ATP and its metabolites are involved in regulating a variety of biological processes including cardiac function, neurotransmission, liver glycogen metabolism, muscle contraction and blood flow [1].

Oral ATP administration has been shown to improve muscular function. Most episodes of lower back pain arise from structures in the lumbar spine, including the paravertebral musculature. ATP is linked to accelerating recovery in people with lower back pain by improving muscular cell function and increased blood flow [2]. Oral ATP administration has been shown to have an early acting effect in sub-acute low back pain and has been approved in France as an adjunct in the treatment of lower back pain [2].

Supplementation of 225 mg per day of enteric-coated ATP supplementation for 15 days resulted in increased total bench press lifting volume as well as within-group repetitions to failure on set one of three with 70% of 1RM [3]. Moreover, 15 days of 400 mg per day of ATP supplementation reduced muscle fatigue and enabled a higher force output during repeated high-intensity bouts of exercise [4]. More recently, 12 weeks of 400 mg of oral ATP disodium salt supplementation in resistance-trained athletes utilizing a periodized resistance-training program (RT) resulted in significant increases in lean body mass, muscle thickness, total strength and vertical jump power [5]. ATP also reduced protein breakdown and limited the loss of strength and power during an overreaching cycle [5].

**Figure 1:**
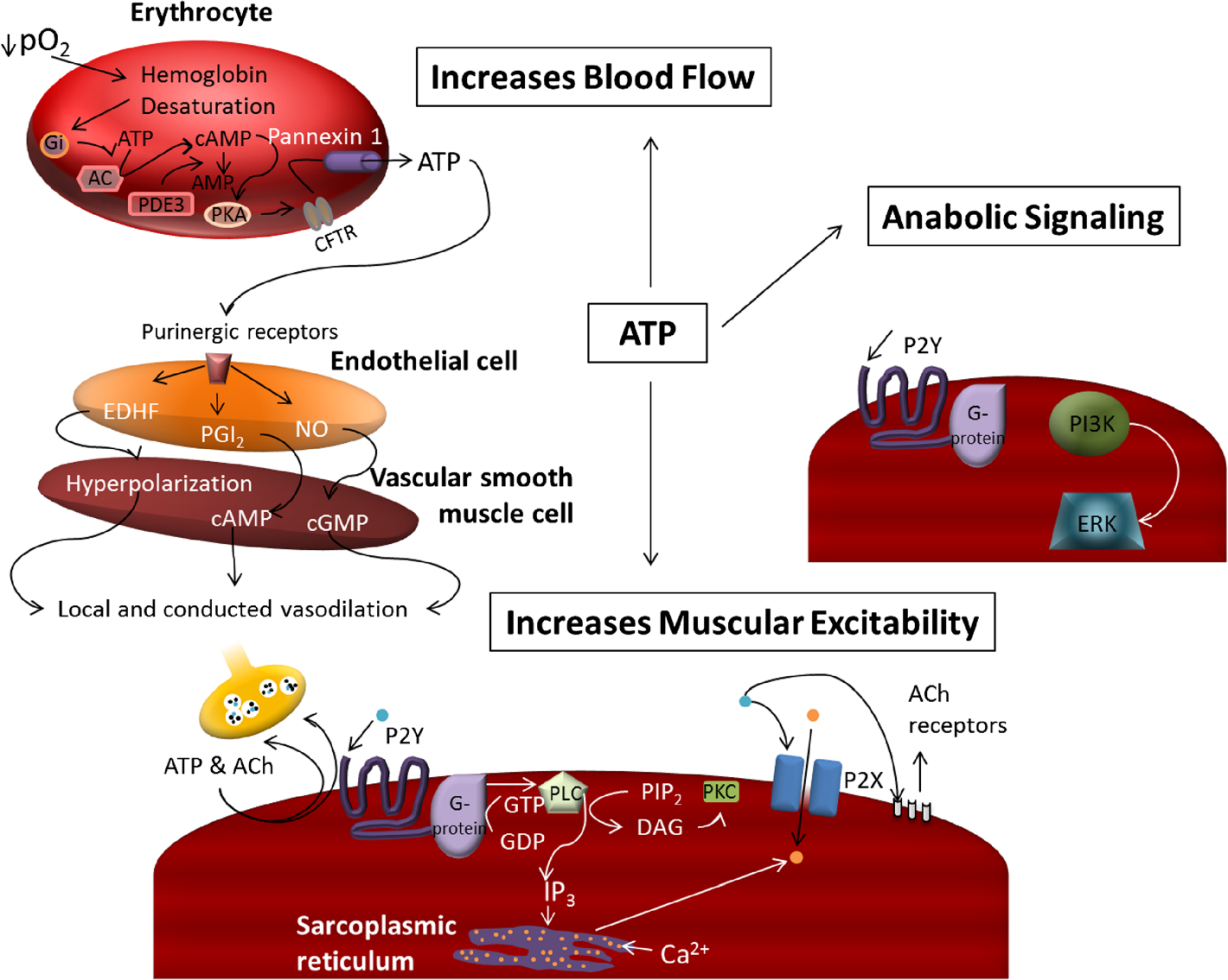
Proposed mechanism-of-action of oral ATP administration.

Three distinct mechanisms-of-action have been proposed for orally administered ATP’s ergogenic benefits: 1) ATP can increase blood flow, resulting in improved oxygen and nutrient delivery to the muscle [5] 2) ATP may increase muscular excitability [6]; 3) ATP can trigger signaling cascades for metabolic adaptation related to neuromuscular activity (phosphorylation of ERK1/2) (see Figure 1) [7]. However, it is unlikely that oral ATP administration will directly increase intramuscular ATP stores.

Erythrocytes function as an oxygen sensor, contributing to the regulation of skeletal muscle blood flow and oxygen delivery, by releasing ATP in proportion to the number of unoccupied oxygen binding sites in the hemoglobin molecule. ATP release results in vasodilation and greater blood flow to the working musculature, thereby enhancing nutrient and oxygen delivery. Thus, during exercise under hypoxic conditions, ATP is released from the red blood cells via pannexin channels. ATP then binds to the purinergic receptors on the endothelial cells [5]. The endothelial cells then produce endothelium-derived hyperpolarizing factor, prostacyclin, and nitric oxide, all of which serve to relax the smooth muscle of the vasculature (see Figure 1) [5]. Infused ATP has been shown to increase blood flow by stimulating endothelial ATP-selective P2Y_2_ receptors and increasing muscle sympathetic vasoconstrictor activity [8]. The vasodilatory and sympatholytic effects of exogenous ATP are mediated via ATP itself rather than its dephosphorylated metabolites [9]. Chronic oral administration of ATP in rats increased portal vein ATP concentration and nucleoside uptake by erythrocytes, which resulted in an increase in ATP synthesis in the erythrocytes [10]. To our knowledge, however, no studies have delineated if oral ATP administration enhances the blood flow response to exercise.

This study used a rat model to examine how different dosages of acute oral ATP administration affected the femoral blood flow response prior to, during, and after an exercise bout. In addition, we performed a single dose chronic administration pilot study in resistance trained athletes.

## Methods

### Animals and experimental protocol

All animal work was conducted in the Department of Biomedical Sciences at the University of Missouri and was approved by the University of Missouri’s Animal Care and Use Committee. Male Wistar rats were obtained from Charles River Laboratory weighing ~250 g. Rats were allowed 7 days to acclimatize to new housing and were maintained on a 12/12-h light/dark cycle, with food (Harlan Laboratories, Tekland Global 14% Rodent Maintenance diet) provided *ad libitum* until the experimental testing day. On the morning of testing, rats had food removed from homes cages at the beginning of the light cycle. Eight hours later, each rat was placed under isoflurane anesthesia and gavage-fed one of the following in 2 ml of water: 3 mg ATP (human equivalent dose of 100 mg), n = 4; 12 mg ATP (human equivalent dose of 400 mg), n = 4; 31 mg ATP (human equivalent dose of 1,000 mg), n = 5; 49 mg ATP (human equivalent dose of 1,600 mg), n = 5 or water only, n = 5 (CTL). All human equivalent doses administered were based upon body surface area conversion factors provided by Reagan-Shaw et al. [11].

Following feeding, a blood flow probe (Transonic Systems, Ithica, NY) was subsequently placed on the proximal portion of the right femoral artery and stimulation electrodes were placed in the right gastrocnemius muscle for an electrically-evoked plantarflexion exercise bout. Blood flow was then monitored continuously: a) 60 min prior to an electrically-evoked leg-kicking exercise (60 V, 100 pps, for 3 min for a total of 180 contractions), b) during the leg kicking exercise, and c) 90 min following exercise. This exercise bout was chosen per previous literature demonstrating that this protocol elicited an increase in femoral blood flow velocity in rats [12].

### Subjects and experimental protocol

All human work was conducted in Department of Health Sciences and Human Performance at the University of Tampa and the protocol was approved by The University of Tampa Institutional Review Board. In a pilot study, 12 resistance-trained male participants (age 23.7 ± 3.6 years; height 179.0 ± 1.0 cm; weight 87.3 ± 6.1 kg) were given 400 mg of ATP as a disodium salt (Peak ATP®, TSI, Missoula, MT) daily 30 minutes before breakfast for 12 weeks. In addition at the beginning of the study and at weeks 1, 4, 8, and 12 subjects consumed the 400 mg of ATP 30 minutes prior to an acute elbow flexor bout (3 sets of 20 contractions at 50% of the subject’s 1-RM). Measurements were taken at weeks 0, 1, 4, 8, and 12. Ultrasonography-determined volumetric blood flow and vessel dilation in the brachial artery [13] was measured at rest before taking the supplement, at rest 30 minutes after supplementation, and then at 0, 3, and 6 minutes after the exercise . An ultrasound Doppler (LOGIQ e 2008, GE Healthcare, Wauwatosa, WI, USA) equipped with an annular phased array transducer probe (12-mm diameter), operating at an imaging frequency of 7.5 MHz and variable Doppler frequencies of 4.0–6.0 MHz, was utilized to measure two-dimensional (2D) brachial arterial diameter and mean blood velocity at rest and following a one arm elbow flexor exercise bout. The depth range of the ultrasound beam was greater than the anatomic location of the brachial artery. Blood flow (Q = vmean · A · 6 × 10^4^, where vmean is mean blood velocity; l/min) was calculated from the amplitude (A) (signal intensity)-weighted, time- and spatial- averaged vmean (m/s), corrected for its angle of insonation, and multiplied by A (m^2^) of the brachial artery. The intraclass correlation coefficient (ICC) for the test–retest of blood flow and brachial arterial diameter ranged from 0.91 to 0.93. The subjects were fully informed of any risks and discomforts associated with the experiments before giving their informed written consent to participate. All subjects worked with a registered dietician and were placed on a diet consisting of 25% fat, 25% protein, and 50% carbohydrates. Inclusion/exclusion criteria indicated that subjects had to have a minimum of 3 years of resistance training experience and could not be taking any nutritional supplements throughout the study. All subjects were told to maintain their normal training volume throughout the study.

### Statistics

For the rat study, a two-way (treatment x time) mixed factorial ANOVA with LSD post hoc analysis was performed to determine if blood flow differed between treatments at each 10-min post-gavage interval. If a significant group, time, or group x time interaction existed the following statistical analyses were performed to further decompose the data: 1) individual independent samples t-tests were performed between treatments at each time point and significance was set at p < 0.01 in order to correct for an inflated type I error rate; 2) dependent t-tests were performed within treatments whereby each time point was compared to the baseline (-60 to -50 min) femoral artery blood flow values. For the rat study, mean femoral artery blood flow areas under the pre-exercise, exercise, post-exercise, and total blood flow curves (AUC) were also computed using SigmaPlot 12.0 which uses the trapezoidal rule algorithm for AUC calculations. Respective AUC values were compared between treatments using one-way ANOVAs with LSD post-hoc analyses where appropriate. All data were expressed as means ± standard error values and significance was set at p < 0.05. For the human data we used a repeated measures analysis of variance using Statistica (StatSoft®, Tulsa, OK, USA) to determine week, time, and week X time effects with an alpha level of 0.05. A tukey post-hoc for pairwise comparisons was run in the event of a significant F-test.

## Results

### Animal data

**Figure 2:**
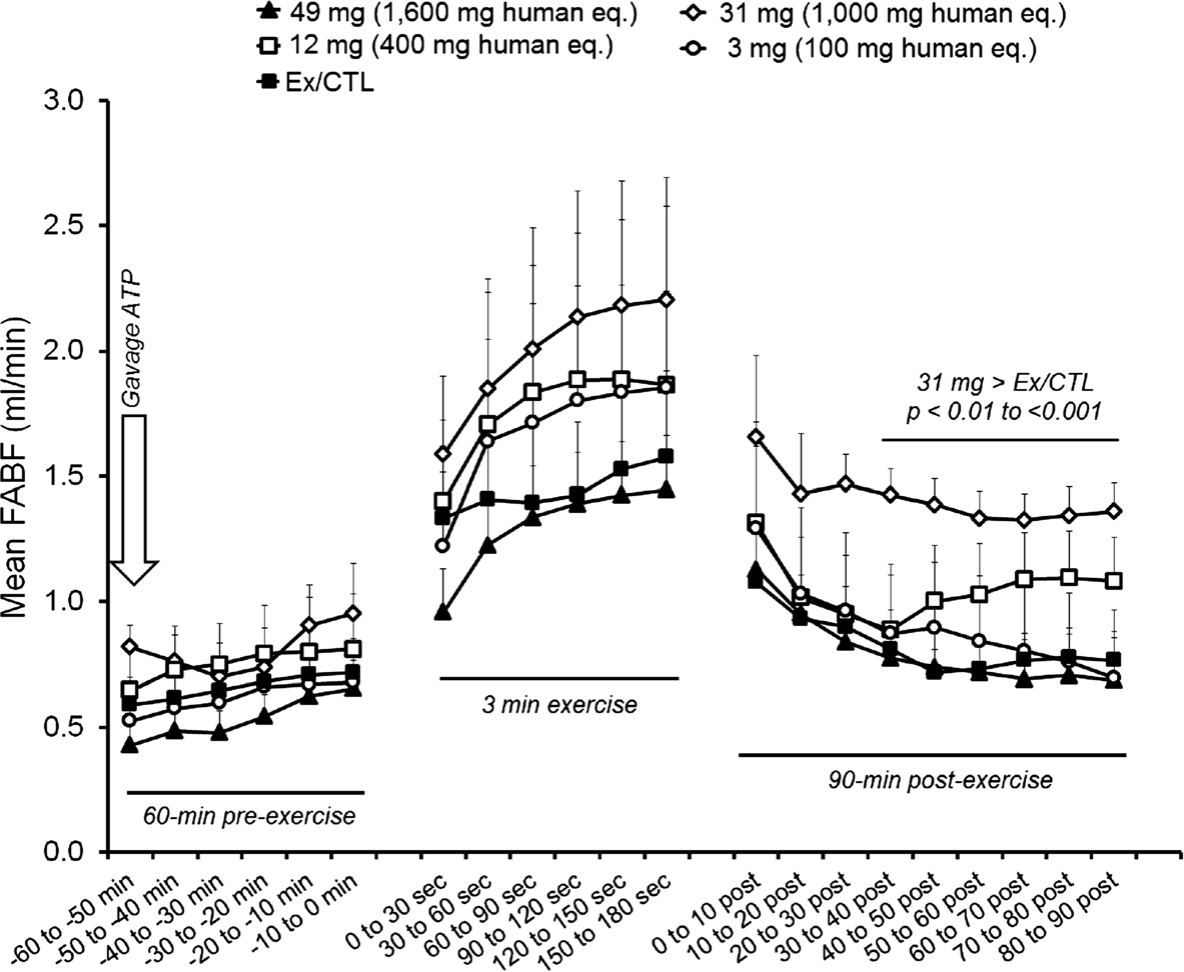
**Mean femoral artery blood flow (FABF) values for 10 min intervals 60 to 0 minutes prior to exercise, during the 3-minute e-stim. exercise bout, and 0 to 90 min following exercise.** Exercise increased blood flow within all groups compared to baseline values. Independent t-tests with correction for multiple comparisons revealed that 31 mg of oral ATP prolonged femoral artery blood flow compared to Ex/CTL rats 30 to 90 min post-exercise (p < 0.01 to p < 0.001). All data are presented as means ± standard errors; n = 4-5 animals per group.

**Figure 3:**
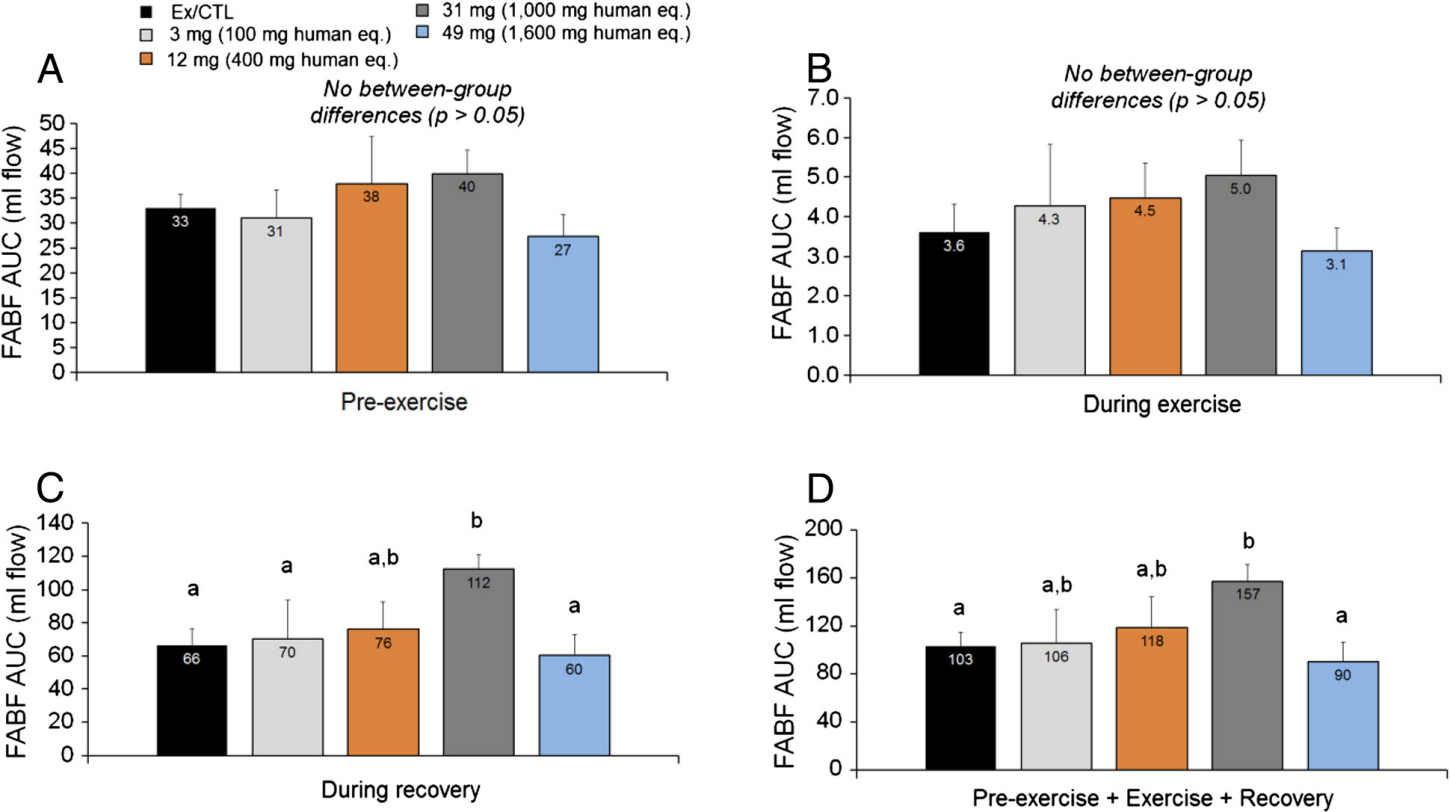
**Mean femoral artery blood flow (FABF) area under the curve (AUC) values prior to exercise (A), during the 3-minute e-stim. exercise bout (B) , during the 90 min recovery period following exercise (C), and during the entire monitoring period (D).** During the recovery period, 31 mg of ATP increased blood flow compared to Ex/CTL, 3 mg, and 49 mg (p < 0.05). During the recovery period, 31 mg of ATP increased blood flow compared to Ex/CTL, 3 mg of ATP, and 49 mg of ATP. During the total monitoring period, 31 mg of ATP increased blood flow compared to Ex/CTL, and 49 mg of ATP. All data are presented as means ± standard errors; n = 4-5 animals per group.

There were significant group (p < 0.001) and time (p < 0.001) effects, though no interaction effect (p > 0.05). When examining within-group differences relative to baseline values, ATP supplementation, independently from the dose, did not increase blood flow pre-exercise. Exercise significantly increased blood flow in all groups at all time points during exercise compared to baseline values within each treatment (p < 0.05). 3 mg ATP had no effect on blood flow during the recovery period. 12 mg ATP (p < 0.001), 31 mg ATP (p = 0.003), and 49 mg ATP (p < 0.001) significantly increased blood flow 0 to 10 minutes post-exercise compared to baseline values within each treatment. In addition, 49 mg ATP significantly increased blood flow 10 to 20, and 20 to 30 minutes post-exercise (p < 0.05) compared to baseline values. Between-group comparisons at each time interval revealed that mean arterial blood flow was elevated in rats fed 31 mg versus Ex/CTL rats at 30 to 90 min post exercise when examining 10-min blood flow intervals (p < 0.01 to <0.001; Figure 2).Rats fed 31 mg demonstrated significantly greater recovery blood flow (p = 0.007) and total blood flow AUC values (p = 0.048) compared to CTL rats (Figure 3).

### Human data

**Figure 4:**
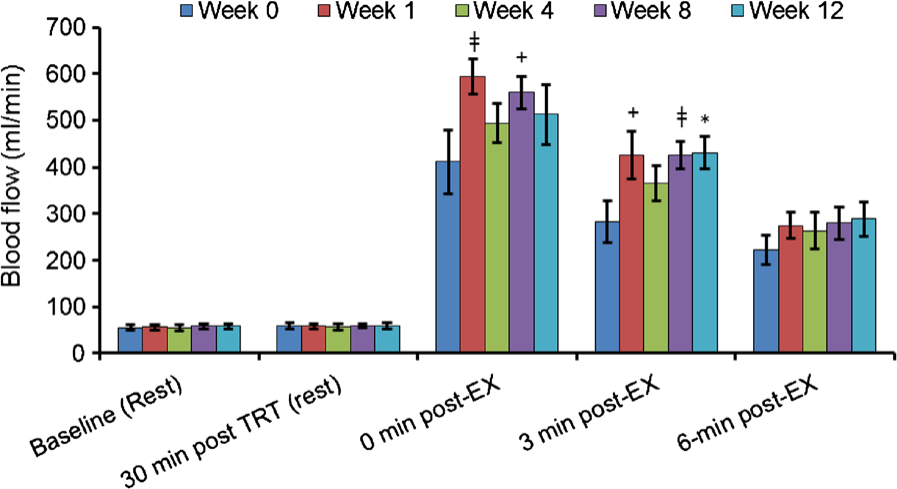
**Changes in brachial blood blow at weeks 1, 4, 8, 12 were compared to control week by a paired *t*-test, **^**‡**^***p*** < 0.01, *p **< 0.05 and **^**+**^***p*** **< 0.10.**

**Figure 5:**
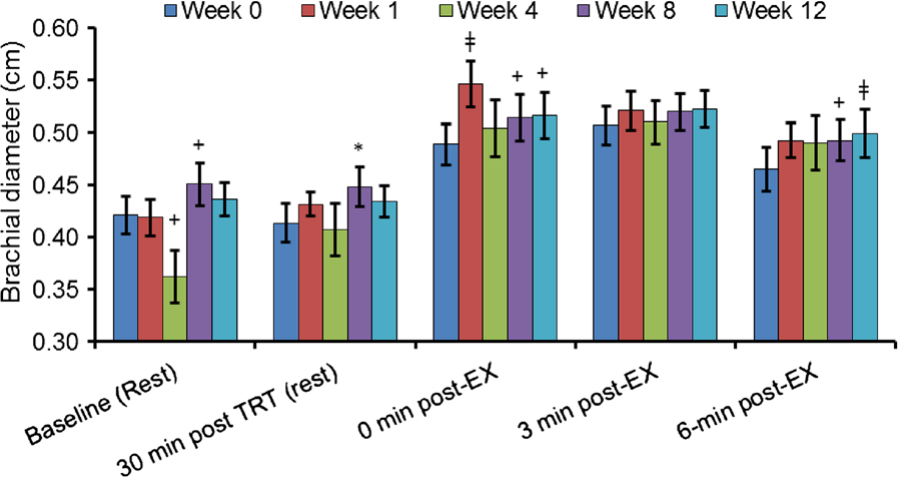
**Changes in Brachial Diameter at weeks 1, 4, 8, 12 were compared to control week by a paired *t*-test, **^**‡**^***p*** < 0.01, *p **< 0.05 and **^**+**^***p*** **< 0.10.**

At week 1 there was significant increase in blood flow at 0 min post exercise (Figure 4; *p* < 0.01) and tended to be increased at 3 min post exercise (p = 0.07) in the ATP supplemented relative to the control week (week 0). This increase in brachial blood flow at week 1 was in conjunction with a significant elevation in brachial dilation at 0 min post exercise (Figure 5; p < 0.01). After 8 weeks of ATP supplementation blood flow tended to be increased at 0 min post exercise (p = 0.07) and was significantly increased at 3 min post exercise at 8 weeks and again at 12 weeks (p < 0.01 and p < 0.05, respectively) relative to the control week. This again was in concurrence with a significant increase in brachial dilation 30 min after ATP treatment at rest at week 8 (p < 0.05) and 6 min post exercise at 12 weeks (p < 0.01) and tended to be increased 0 post exercise at 8 and 12 weeks (p < 0.10) relative to the control week.

## Discussion

Wilson et al. recently suggested that oral ATP supplementation can significantly impact athletic performance, skeletal muscle hypertrophy and recovery; however, the study did not utilize methodologies to investigate the potential mechanism for the observed ergogenic effects [6]. One of the proposed mechanisms of action of oral ATP administration is an increase in blood flow, resulting in improved oxygen and nutrient delivery to the muscle. Enhanced blood flow to an exercising skeletal muscle is expected to improve removal of metabolic waste products such as lactate and urea. Following exercise nutrient delivery and cell swelling play a vital role in the skeletal muscle adaptation response. Improvements in blood flow conceivably would allow for greater delivery of nutrients for skeletal muscle repair following a muscle damaging bout of training resulting in increases in muscle hypertrophy previously seen with oral ATP administration. The main finding of this study was that orally administered ATP as a disodium salt indeed increases blood flow in exercising animals and humans, most prominently during the recovery period from exercise. Significant improvements could be measured at a daily dose of 400 mg ATP in as little as one week in the human study.

Though the exact mechanism of oral ATP absorption is currently not fully understood, animal studies have shown that the chronic oral administration of ATP resulted in measurable changes in muscle metabolism, peripheral blood flow, and blood oxygenation [10,14] and human studies have resulted in significant improvements in body composition and performance [4,6]. Studies on the oral availability of ATP showed that it is unlikely that oral ATP administration will directly increase intramuscular ATP stores as a single dose of orally administered ATP in humans did not increase ATP concentrations in blood [15]. The measurement of circulating free plasma ATP derived from oral ATP supplementation is very unlikely because exogenous free ATP is rapidly taken up by blood components or is rapidly metabolized. Kichenin et al. showed, in rats, that chronic oral administration of ATP increased portal vein ATP concentration and nucleoside uptake by erythrocytes, which resulted in an increase in ATP synthesis in the erythrocytes [10]. In other animal studies the administration of oral ATP resulted in a rapid degradation through ectonucleotidase triphosphatase diphosphohydrolase 1 (CD39), present on the luminal side of intestinal enterocytes, which dephosphorylate ATP via ADP to AMP, after which ecto-5′-nucleotidase (CD73) degrades AMP to adenosine [16,17]. Following absorption of adenosine and inorganic phosphate in the small intestine and the portal circulation these moieties are then incorporated into liver ATP pools, leading to expansions of these pools. Therefore, the systemic and oral administrations of ATP result in the expansions of liver, blood (red blood cells) and blood plasma (extracellular) pools of ATP which were shown for the first time by Rapaport et al. [18,19].

Blood flow during exercise is indicative of nutrient (amino acids, glucose, etc.) and oxygen delivery rate. As such, increased blood flow will indicate greater nutrient availability for the working musculature, and, in theory, the muscle should have the capacity to recover more quickly between sets, maintain performance longer, and repair microtrauma more efficiently between training sessions. Wilson et al. [6] hypothesized that the observed increases in lean body mass, markers of athletic performance, and resistance to an overreaching status with chronic ATP supplementation were due to enhanced blood flow leading to enhanced recovery, although this remained to be directly examined until the current study. However, despite increased blood flow during ATP infusion, oxygen consumption does not increase [20]. Considering these two studies, it is possible that ATP is more efficacious for anaerobic versus aerobic based exercise. However, ATP’s efficacy in an endurance model remains to be investigated. Likewise, the exact mechanism whereby ATP increases post-exercise blood flow also remains to be determined, although others have hypothesized that this may be due to: a) ATP degradation products being taken up by erythrocytes and resynthesized into ATP; b) vasodilation of ATP degradation (i.e., adenosine) products; and/or c) adenosine-stimulated nitric oxide and prostacyclin synthesis and downstream signaling [4].

L-citrulline or L-arginine are amino acid precursors to nitric oxide and have been marketed as potential ergogenic aids based on their ability to increase blood flow to the exercising muscle. However, the daily dose needed to increase blood flow is high (6-24 g) and the ergogenic response may depend on the training status and health of the subjects [21]. Whereas some studies involving untrained or moderately healthy subjects showed that nitric oxide donors could improve tolerance to aerobic and anaerobic exercise, no significant improvements were measured in healthy [22] or highly-trained subjects [21,23]. In contrast, oral ATP increases blood flow at mg doses and has been shown to increase lean body mass, strength and power in highly trained individuals [6]. Therefore, oral ATP supplementation is an apparently efficacious method if the intent is increasing post-exercise blood flow and nutrient delivery.

Limitations of this study include the lack of control group and a rigorous control of other potentially confounding variables such as potential differences in exercise habits or baseline dietary habits and dietary supplement use in the human data. We propose future research to assess the effects of oral ATP administration on blood flow in a placebo-controlled crossover or parallel design.

## Conclusion

Oral ATP administration can increase blood flow, and this effect is particularly prominent following exercise. Increased blood flow due to ATP supplementation may be the mechanism responsible for ergogenic effects following chronic ATP supplementation as previously reported in the scientific literature. However, the exact mechanism whereby ATP increases blood flow during post-exercise recovery periods remains unknown and future investigation in this area is warranted.

## Competing interests

JMW, JMJ, RPL, MDR, and CLC declare no competing interests. JR is employed by Metabolic Technologies, Inc. which engages in business trade with TSI (USA), Inc. RJ and MP are, and CML was a consultant of TSI, Inc.

## Authors’ contribution

The manuscript was written through contributions of all authors. All authors have given approval to the final version of the manuscript.
